# Spread-out Bragg peak FLASH: quantifying normal tissue toxicity in a murine model

**DOI:** 10.3389/fonc.2024.1427667

**Published:** 2024-07-03

**Authors:** Line Kristensen, Per Rugaard Poulsen, Eleni Kanouta, Sky Rohrer, Christina Ankjærgaard, Claus E. Andersen, Jacob G. Johansen, Yuri Simeonov, Uli Weber, Cai Grau, Brita Singers Sørensen

**Affiliations:** ^1^ Danish Centre for Particle Therapy, Aarhus University Hospital, Aarhus, Denmark; ^2^ Department of Experimental Clinical Oncology, Aarhus University Hospital, Aarhus, Denmark; ^3^ Department of Clinical Medicine, Aarhus University, Aarhus, Denmark; ^4^ DTU Health Tech, Technical University of Denmark, Roskilde, Denmark; ^5^ Institut für Medizinische Physik und Strahlenschutz, Technische Hochschule Mittelhessen, Giessen, Germany; ^6^ Department for Biophysics, GSI Helmholtzzentrum für Schwerionenforschung, Darmstadt, Germany

**Keywords:** FLASH radiation, spread-out Bragg peak, normal tissue sparing, acute toxicity, late toxicity

## Abstract

**Objective:**

A favorable effect of ultra-high dose rate (FLASH) radiation on normal tissue-sparing has been indicated in several preclinical studies. In these studies, the adverse effects of radiation damage were reduced without compromising tumor control. Most studies of proton FLASH investigate these effects within the entrance of a proton beam. However, the real advantage of proton therapy lies in the Spread-out Bragg Peak (SOBP), which allows for giving a high dose to a target with a limited dose to healthy tissue at the entrance of the beam. Therefore, a clinically relevant investigation of the FLASH effect would be of healthy tissues within a SOBP. Our study quantified the tissue-sparing effect of FLASH radiation on acute and late toxicity within an SOBP in a murine model.

**Material/Methods:**

Radiation-induced damage was assessed for acute and late toxicity in the same mice following irradiation with FLASH (Field dose rate of 60 Gy/s) or conventional (CONV, 0.34 Gy/s) dose rates. The right hindleg of unanesthetized female CDF1 mice was irradiated with single-fraction doses between 19.9-49.7 Gy for CONV and 30.4-65.9 Gy for FLASH with 5-8 mice per dose. The leg was placed in the middle of a 5 cm SOBP generated from a mono-energetic beam using a 2D range modulator. Acute skin toxicity quantified by hair loss, moist desquamation and toe separation was monitored daily within 29 days post-treatment. Late toxicity of fibrotic development measured by leg extendibility was monitored biweekly until 30 weeks post-treatment.

**Results:**

Comparison of acute skin toxicity following radiation indicated a tissue-sparing effect of FLASH compared to conventional single-fraction radiation with a mean protection ratio of 1.40 (1.35-1.46). Fibrotic development similarly indicated normal tissue sparing with a 1.18 (1.17-1.18) protection ratio. The acute skin toxicity tissue sparing was similar to data from entrance-beam irradiations of Sørensen et al. (4).

**Conclusion:**

Full dose-response curves for acute and late toxicity after CONV and FLASH radiation were obtained. Radiation within the SOBP retains the normal-tissue-sparing effect of FLASH with a dose-modifying factor of 40% for acute skin damage and 18% for fibrotic development.

## Introduction

Radiotherapy is a constant balance between enough radiation to cure the cancer and avoiding lethal toxic side effects to healthy tissues. As toxicity to healthy tissues is a limiting factor in radiotherapy, reducing side effects is a crucial aim of novel approaches.

FLASH irradiation has shown promise in preclinical studies to improve treatment outcome by reducing side effects. With FLASH, the dose is delivered ultra-fast, which has been demonstrated to reduce healthy tissue toxicity while maintaining the curative effect on cancer (1–9). The reduced healthy tissue toxicity compared to conventional dose rates, the FLASH effect, has been documented in several tissues *in vivo* for x-ray (10–13), electron (1, 2, 8, 9, 14, 15) and proton irradiations (3–5, 16, 17).

Proton irradiation has particular potential, compared to photons and electrons, due to its depth-dose distribution (18), but few FLASH studies use this ability. Utilizing a spread-out Bragg peak (SOBP) to create a plateau of high dose is the primary advantage of proton therapy in clinical practice (19). This method results in low dose to the entrance of the irradiated tissue, high dose to deep-seated tumors within the SOBP, and no dose behind (19). Reducing the integral dose to healthy tissues can cause less damage and, therefore, fewer side effects. Despite this, proton FLASH studies have predominantly focused on the entrance region of the beam (6), due to technical limitations.

The limitations for generating a FLASH SOBP lie in two main factors. The typical clinical way of forming an SOBP is by using several energy layers with energies adapted to the tumor depth. When working with FLASH, requiring several energy layers is problematic. Cyclotron-based facilities can only deliver ultra-high dose rates at the highest proton beam energies, with large beam ranges of 30-40 cm. Furthermore, the delivery of multiple energy layers would prolong the treatment duration due to beam pauses for each energy shift, which could be detrimental to the FLASH effect. Thus, a passive SOBP generation is needed to obtain FLASH conditions.

A proton SOBP can be obtained from a mono-energetic beam by using a range modulator to broaden the energy distribution. This approach enables the combination of FLASH and SOBP (17, 20–22), which could improve the radiotherapeutic treatment (23–25). The dose conformality of the SOBP would ensure low doses to most healthy tissues. At the same time, the FLASH effect would reduce toxicity in the irradiated healthy tissues, resulting in fewer side effects overall. The limited literature in this field suggests retained neuroprotection and abdominal sparing (26, 27), yet quantification of the combined SOBP-FLASH effect on tissue toxicity is very sparse.

In order to investigate side-effect reduction by FLASH in the clinical practice of SOBP, the current study was designed to quantify the tissue-sparing effect of SOBP proton FLASH irradiation. Simultaneously, the study was designed to enable direct comparison to entrance-beam proton FLASH effects published in Sørensen et al. (4). Using a murine model, we constructed full dose-response curves of biological response for conventional and FLASH dose rates through two simultaneous assays to capture the full impact of the treatment. Initially, acute radiation-induced damage was evaluated using a skin damage assay, followed by an assessment of late radiation-induced damage using a fibrotic assay, both conducted on the same animals.

## Methods

### Mouse preparation

Female C3D2F1 twelve to eighteen weeks old mice were used for normal tissue damage assessment. The mice were obtained from Janvier Labs (Le Genest-Saint-Isle, France) and housed in groups of four with weights evenly distributed between cages. The mice were provided food pellets and water ad libitum and acclimated to our lab for at least six weeks. All experiments were performed under ethical and legal permit from the Danish Licensing Authority no. 2022-15-0201-01110, and the study adheres to the ARRIVE guidelines (28). Mice were weighed, earmarked, and given an ID shortly before irradiations.

Mice were allocated to first a dose rate, then to different doses, resulting in a total of 19 treatment groups (**Table 1**). Allocation to treatment groups was partly randomized. However, it was manually ensured that there was not more than a 10 Gy difference between mice in the same cage due to welfare considerations. Treatment groups consisted of doses between 19.9 - 49.7 Gy for conventional radiations with low dose rate (CONV) and doses between 30.4 - 65.9 Gy for ultra-high dose rate radiations (FLASH). Based on previous data, the dose groups were chosen to provide full dose-response curves for both acute and late responses (3, 4).

**Table 1:** Mice per treatment group included in analysis of skin toxicity and fibrosis.

FLASH			CONV		
Dose (Gy)	Skin toxicity assay	Fibrosis assay	Dose (Gy)	Skin toxicity assay	Fibrosis assay
30.4	7	7	19.9	7	7
35.5	7	7	24.9	8	8
40.5	8	8	29.8	8	8
43.1	8	8	32.3	7	7
45.6	8	8	34.8	8	8
48.1	8	8	37.3	8	8
50.7	8	8	39.8	8	6
55.7	7	7	44.8	8	8
60.8	7	7	49.7	5	5
65.9	5	4			
Total	73	72	Total	67	65

Irradiations were conducted over nine months in four consecutive experiments: three with 40 mice and one with 25 mice. Each mouse was considered an experimental unit, giving 145 units. The sample size was based on previous studies of similar designs (3, 4). Irradiations were conducted at the same time interval during the day (between 5 PM and 12 AM) to avoid the influence of diurnal rhythm.

The study design did not enable investigators to be blinded during irradiations due to the apparent differences in delivery time between CONV and FLASH. Likewise, dose rates could not be fully randomized within the same day due to the resource-intensive task of changing between CONV and FLASH beams combined with the limited time available with beamline access. Instead, within the same day, irradiations used either CONV first, and FLASH thereafter, or vice versa. The order of treatment doses was randomized within the CONV and FLASH groups.

### SOBP irradiations

**Figure 1:**
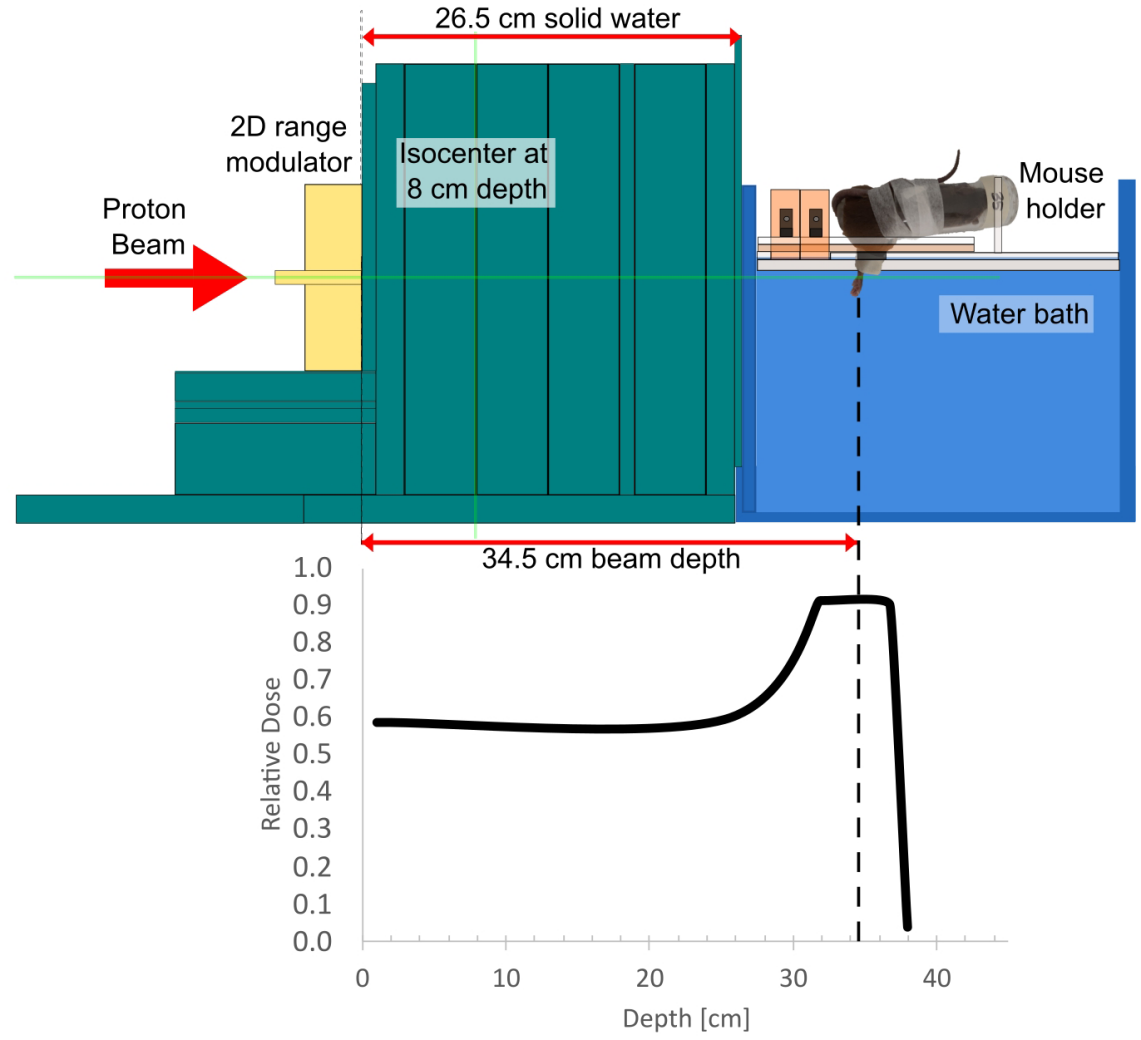
A schematic of the experimental setup for FLASH irradiations and the placement of the mouse foot relative to the generated spread-out Bragg peak. The treatment depth was 34.5 cm for FLASH (250 MeV) and 33 cm for CONV (244 MeV).

The effect of FLASH irradiation within an SOBP was quantified using an experimental setup similar to previous FLASH studies with proton entrance beams (3, 4). Like these previous studies, irradiations used a fixed horizontal proton beamline (ProBeam, Varian, a Siemens Healthineers Company, Palo Alto, CA, USA) at the Danish Centre for Particle Therapy (Aarhus University Hospital, Denmark). A 2D range modulator (20–22) designed and manufactured at the University of Applied Sciences in Giessen, was used to generate a 5 cm wide SOBP from a mono-energetic beam of 250 MeV for FLASH and 244 MeV for CONV. A beam degrader of 26.5 cm solid water was used in front of a water bath in which the mouse leg target was placed in total treatment depths of 34.5 cm for FLASH and 33 cm for CONV (**Figure 1**).

The pencil-beam scanning (PBS) proton irradiations followed a spot pattern of 5 x 7 spots with a spot spacing of 6 mm in the isocenter plane and 6.5-6.8 mm in the treatment depth, yielding a field size of approximately 2 x 3 cm (**Supplementary 1A**) to cover the mouse foot, as described further in Kanouta et al. (29). The spots at the rim of the field had 40% higher weight than those in the inner part. For CONV, to achieve a low field dose rate of 0.35 Gy/s and to increase motion robustness, the spot pattern was delivered with 144-360 repaintings (depending on the dose) and mean beam pauses of 4.4 ms between each spot delivery. For FLASH, the spot pattern was painted once to achieve a field dose rate of approximately 60 Gy/s. Daily fluctuations in the current output of FLASH irradiations were accounted for by adapting the requested beam current to give the intended field dose rate. A field dose rate of 60 Gy/s was chosen rather than using the maximum possible beam current because this allowed better adaptation to daily variations and because entrance-beam FLASH experiments with the same murine model indicated that a maximum FLASH level was reached at this field dose rate (30). Both the field dose rates and beam energies were chosen to resemble previous proton FLASH studies (3, 4).

The target was the right hind leg of unanesthetized mice restrained in Lucite jigs. The mice were placed in a water bath of 25°C with the leg extended into the water and brass shielding of the mouse body (**Figure 1**). One mouse was radiated per round.

### Dosimetry

Several dosimetric methods were implemented before and during treatment to ensure the correct delivery of the planned dose to the target. Radiochromic films (EBT-XD Gafchromic films) were used to measure the dose profile at the treatment depth. An Advanced Markus ionization chamber was used for depth-dose characterization and absolute dose calibration. The field dose rate was calculated for each mouse irradiation as the dose divided by the total field duration. For FLASH, the field duration was extracted as the sum of all spot deliveries in machine log files (31). For CONV, the field duration was measured with fiber-coupled scintillators since the machine log files lacked information on the beam pauses between spots (31). Similar to Sørensen et al. (4), the local PBS dose rate DR_PBS95%_ was calculated in the plane of the mouse leg target as 95% of the dose divided by the time interval between the cumulative dose reached 2.5% and 97.5% of its final value (32).

To catch gross errors, *in vivo* dosimetry was performed using alanine dosimeters wrapped in parafilm and placed in the water bath by the mouse foot. Alanine pellets (Batch CD600 from Harwell Dosimeters) with a diameter of 4.8 mm and a height of 2.8 mm were read out as described previously (33). A beam quality correction factor of kQ = 1.032 ± 0.021 was applied to correct for the change in alanine response in the proton beam SOBP relative to the response in the ^60^Co calibration beam. The correction was derived based on a direct comparison between alanine measurements and measurements with a small-core graphite calorimeter (34).

### Normal tissue damage assays

The acute biological response to radiation was quantified using an acute skin toxicity assay. The assay visually assessed skin damage using an established skin damage scoring system (35, 36). Skin damage was quantified with a grade between 1.5 (mild damage) and 3.5 (severe damage) with increments of 0.5. The grade was based on the following parameters on the foot alone: skin redness, percentage area with hair loss and moist desquamation, foot form and number of visibly distinguishable toes.

To achieve a more complete understanding of the effect of FLASH, late damage was assessed on the same mice after developing acute damage. The late biological response in the radiated leg was quantified using a leg extension assay, which is a functional assay for the level of subcutaneous fibrosis (36, 37). The assay visually assessed leg flexibility by extending the leg without force. The fibrosis was quantified as 50%, 25% or 0% flexibility by grades 2, 3, or 4, measured relative to the non-irradiated leg (36, 37). For images of the extension assay, see supplementary in Overgaard et al., 2023 (36).

One of three observers scored the toxicity and photographically documented the acute damage when feasible. Observers scored the mice while blinded for treatment and previous grades and with minimal interobserver variability (38). Each mouse was assessed for acute response daily between eight to twenty-eight days post-irradiation to ensure that the maximum reached damage was captured. For late response, the mice were assessed biweekly between 9-30 weeks for time-resolved fibrotic development. Each mouse assessment used the non-irradiated left hind leg as a control to the irradiated right hind leg. When determining a parameter in the assay, e.g. in terms of percentage hair loss on the foot, the non-irradiated left foot was used as reference to the ‘normal’ hairiness for that specific mouse, thus utilizing the mouse to be its own control in both assays. The grades provided data on the damage development over time and captured the maximal damage for each mouse.

### Analysis of biological response

Maximal damage was analyzed as a function of dose in dose-response curves for FLASH and CONV. As the acute toxicity assay includes five toxicity grades of interest (Grade 1.5 - 3.5), and the fibrotic assay includes three (Grade 2 - 4), a separate graph was made for each grade. Within each grade, the scores for each mouse were converted to binomial data, informing whether the grade was achieved at least once. Logistic regression was used to model the toxicity as a function of dose for FLASH and CONV, as in Sørensen et al. (3, 4).

An automated code was used for data management, including relating mouse ID with treatment and data analysis to ensure investigator blinding during analysis. Mice were excluded based on predefined criteria of humane endpoints: weekly weight loss above 20%, skin damage outside the target area, development of severely necrotic tissue or if alanine dosimetry indicated a delivered dose that differed more than 15% from the planned dose. Data management, analysis and visualization used the statistical program R Studio and the graphical program GraphPad Prism (39, 40).

Dose-response curves were used to quantify the effect of the two treatments (CONV and FLASH). The FLASH effect was quantified for all toxicity grades as the dose-modifying factor (DMF), that is, the ratio between CONV and FLASH at TD_50_ (median toxic dose: Dose at which toxicity occurred in 50% of cases) and their corresponding 95% confidence intervals. A DMF below 1 would indicate increased toxicity from the FLASH treatment compared to CONV, and likewise, a factor above 1 would indicate a decreased toxicity and, thus, an improved radiotherapeutic treatment.

The resulting acute dose-response curves and DMF were compared to the data published in Sørensen et al. (4), to enable a comparison of the FLASH effect between entrance-beam and SOBP proton irradiation. Sørensen et al. and the current study used the same acute toxicity assay, murine model, homogenous dose field size and experimental setup with horizontal beam line and water bath (4). The two studies differed slightly in proton spot spacing, spot weighing, and FLASH field dose rate.

## Results

### SOBP acute response

**Figure 2:**
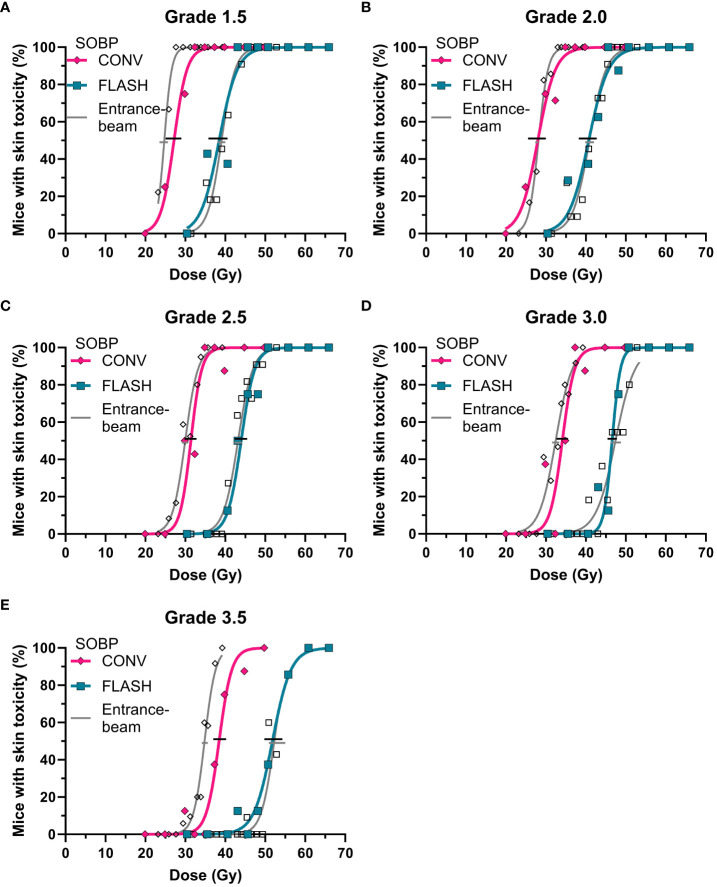
Dose-response curve for acute toxicity after irradiations with conventional (CONV, pink diamonds) or FLASH (blue squares) dose rates compared to previously published data on entrance-plateau irradiation-induced toxicity (grey) (4) for **(A)** grade 1.5, **(B)** grade 2.0, **(C)**, grade 2.5, **(D)** grade 3.0, and **(E)** grade 3.5.

The acute toxicity resulted in complete dose-response curves ranging from 0% to 100% responders for both CONV and FLASH dose rates, illustrated in **Figure 2**. The FLASH dose-response curve consistently required higher doses for a response than CONV, which is evident across all toxicity grades (**Figure 2**). Each treatment group had 5-8 mice (**Table 1**). Five mice were excluded from the data analysis: one due to a technical error, two due to off-target toxicity, and two due to low alanine doses, although there were no other indications of erroneous dose delivery. For the remaining mice, the mean (SD) alanine dose relative to the planned dose was 97.1% (2.1%) for CONV and 97.7% (2.7%) for FLASH (**Supplementary 2**). The quantified difference between the curves, the DMF, was similar across all toxicity grades, with an overall mean of 1.40 (1.35-1.46), but tended to be smaller with more severe grades (**Table 2**).

**Table 2:** Dose-modifying factor (DMF) and TD_50_ (toxic dose for 50% of the population) mean (95% CI) for acute toxicity grades.

	Mean (95% CI)	Grade 1.5	Grade 2.0	Grade 2.5	Grade 3.0	Grade 3.5
**SOBP**	TD_50_ CONV	27.1 (25.0-29.0)	28.0 (25.5-30.0)	31.4 (29.8-32.8)	34.1 (32.6-35.5)	38.4 (37.0-40.2)
	TD_50_ FLASH	38.4 (35.8-40.5)	40.7 (38.2-42.7)	44.0 (42.3-46.5)	46.6 (45.4-47.8)	51.8 (49.8-54.3)
	**DMF**	1.42 (1.33-1.62)	1.46 (1.36-1.67)	1.40 (1.34-1.53)	1.37 (1.31-1.47)	1.35 (1.29-1.47)
**Entrance**	TD_50_ CONV	24.7 (23.5-25.6)	28.1 (27.3-28.1)	30.0 (29.1-30.7)	32.4 (31.5-33.3)	34.8 (34.1-35.6)
	TD_50_ FLASH	39.1 (38.0-40.0)	40.9 (39.8-42.0)	43.2 (42.2-44.1)	47.4 (48.3-50.9)	52.2 (50.9-55.0)
	**DMF**	1.58 (1.48-1.70)	1.45 (1.38-1.54)	1.44 (1.37-1.52)	1.46 (1.45-1.61)	1.50 (1.43-1.61)

The data on entrance-beam irradiations are directly from Sørensen et al. (4).

The mean (SD) of the field dose rate was 0.34 Gy/s (0.04 Gy/s) for CONV and 60.0 Gy/s (4.2 Gy/s) for FLASH (**Supplementary 3**). For a FLASH delivery with a field dose rate of 60 Gy/s, the mean DR_PBS95%_ in the high dose region that received 95% or higher doses was 86 Gy/s (range: 72-101 Gy/s) (**Supplementary 1B**). For CONV, DR_PBS95%_ was essentially equal to the field dose rate due to the repainting.

Acute skin damage was induced around day 12 in a dose- and dose-rate-independent manner (**Supplementary 4**). For CONV and FLASH, the maximum impact was reached around Day 14 - 17 post-irradiation (**Supplementary 4**). The maximal median score and the total time spent with each score were dose-dependent. The skin damage had a similar time pattern for CONV and FLASH when considering the FLASH factor's effect in the dose dependency (**Supplementary 4**).

### Comparison of SOBP and entrance acute response

The same acute dose-response curves were used to compare the FLASH dose modification in entrance-proton irradiations of Sørensen et al. (4) (**Figure 2**). The biological response to FLASH was similar between entrance-beam and SOBP irradiations, a trend seen for all toxicity grades (**Figure 2**). There was, however, a discrepancy between the CONV response in the entrance and the SOBP, with a generally lower dose needed to achieve toxicity in the entrance study. The ratio of TD_50_ between the SOBP (current study) and the entrance plateau (4) across the five toxicity levels was 1.06 (95% CI: 0.99-1.1) for CONV and 0.99 (95% CI: 0.98-1.02) for FLASH (**Table 2**). The higher TD_50_ for CONV in the SOBP is reflected in a lower DMF compared to those reported for entrance-beam irradiations (4).

### SOBP late response

**Figure 3:**
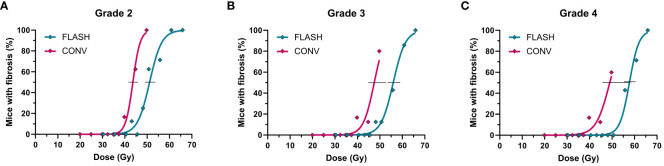
Dose-response curve for late toxicity after irradiations with conventional (pink) or FLASH (blue) dose rates for a fibrotic score **(A)** grade 2, **(B)** grade 3 and **(C)** grade 4.

Similarly to the acute skin response, the late toxicity differed between the FLASH and CONV dose rates (**Figure 3**), with a mean DMF of 1.18 (1.17-1.18) (**Table 3**). In addition to the mice excluded prior to acute toxicity analysis, three mice were excluded from the late toxicity analysis due to necrotic development before a fibrotic score was reached (**Table 1**). The figure demonstrates a full dose-response curve for the moderate fibrotic development equal to grade 2, with a clear difference between FLASH and CONV, while the other grades showed similar results but lacked completion of the CONV curve, resulting in the overlapping 95% confidence interval. (**Table 3**; **Figure 3**).

**Table 3:** Dose-modifying factor (DMF) for late toxicity grades.

Mean (95% CI)	Grade 2	Grade 3	Grade 4
TD_50_ CONV	43.6 (41.6-45.9)	47.5 (44.8-52.7)	49.0 (45.6-58.2)
TD_50_ FLASH	51.1 (49.0-53.7)	55.9 (53.4-59.1)	57.7 (55.3-60.5)
DMF	1.17 (1.07-1.29)	1.18 (1.01-1.32)	1.18 (0.95-1.33)

## Discussion

FLASH irradiations in the SOBP retained the tissue-sparing effect, highlighting its potential to improve side effects in current clinical practice. Using a 2D range modulator to form an SOBP with a FLASH dose rate, we saw a reduced biological response to radiation for both acute (**Figure 2**) and late damage (**Figure 3**). Thus, the FLASH SOBP treatment was less toxic than the conventional dose rate. The different toxicity grades all showed the same trend, confirming the robustness of our assay (**Figures 2**, **3**). The use of FLASH in the SOBP showed a clear change in the biological response of the irradiated foot.

One of the major limiting factors of radiotherapy is the healthy tissue toxicity, and while some toxicity is expected, reduction is the key to improving treatment. Our study had a mean acute skin-sparing effect of 1.40 (range 1.35-1.46), meaning that a 40% (35-46%) higher dose was needed for FLASH to cause the same degree of acute skin damage as for a conventional dose rate (Specific effect for each toxicity grade in **Table 2**). Compared to the reported abdominal LD_50_-derived (lethal dose for 50%) FLASH effect in Evans et al. (27) of 10-20%, our acute skin TD_50_-derived FLASH effect is markedly higher, possibly due to differences in sparing effects across different tissue types (41). Our results on acute damage validate the sparse literature on the presence of FLASH effects in SOBP (26, 27).

Another impact of radiation treatment is the late damage, which gives continued side effects long after the treatment. FLASH needed an 18% (17-18%) higher dose than the conventional dose rate to cause the same radiation-induced fibrosis. Thus, our FLASH treatment had a lower sparing effect for late relative to acute effects. The lower late effect suggests that late-responding tissues are less responsive to the FLASH effect, as indicated in previous studies (3, 42, 43). Studies quantifying the FLASH effect in late-responding tissues are, however, sparse, making it difficult to compare our effect with other studies and tissues (41). Compared to a tumor control study using the same murine model and fibrotic assay with entrance-proton irradiations (3), our SOBP study showed a similar mean DMF for grade 3 radiation-induced fibrosis of 1.18 relative to their 1.14 (3). Likewise, their fibrotic grade 3 TD_50_ (48.6 Gy for CONV, 55.6 Gy for FLASH (3)) was very similar to ours (47.5 Gy for CONV, 55.9 Gy for FLASH). Despite the entrance-beam study having a low and skewed distribution of animals per dose group (3), which does not support a robust dose-response curve, the fibrotic DMF also seems alike between entrance and SOBP irradiations. Tissue sparing was thus present for acute skin damage and radiation-induced fibrosis, validating the FLASH effects in SOBP and demonstrating a retained effect compared to entrance-proton irradiation.

The majority of proton FLASH studies so far have focused on the entrance plateau rather than the SOBP (6). Therefore, our study design directly compared one previous study in the entrance plateau from Sørensen et al. (4) and our new study in the middle of an SOBP. The acute FLASH effect was retained when moving from the entrance plateau to SOBP, as seen from the overlapping dose-response curves (**Figure 2**). The comparison of entrance and SOBP-induced acute toxicity did indicate slight differences in the FLASH effect (DMF in **Table 2**). However, these were due to differences between the two conventional treatments (TD_50_ in **Table 2**). The conventional entrance plateau treatment was more radiosensitive than the SOBP treatment. The CONV dose rate was very similar between the two studies, and the difference cannot be explained by differences in linear energy transfer (LET), as the SOBP treatment would be expected to be more radiosensitive. We do not have a clear explanation for the observed difference in the CONV dose-response curves. Overall, our findings support that the tissue sparing of proton FLASH is unaffected by treatment depth in the beam (26, 27, 44), and thus, the beneficial ability of SOBP does not compromise the FLASH effect.

The similarity between entrance and SOBP was the same across all toxicity grades, indicating that at the same dose rate and within a limited span of very high single doses, the FLASH effect is not influenced by the dose dependency of our endpoints. Conversely, the FLASH effect was shown to be dose-rate dependent in a recent study using the same acute toxicity assay (30).

While the field dose rate for FLASH (49-70 Gy/s, **Supplementary 3**) was comparable with the entrance-beam FLASH study of Sørensen et al. (65-92 Gy/s (4)) the much broader proton spots in the SOBP depth (~8mm (29) versus ~4 mm (4, 45)) resulted in considerably smaller PBS dose rates (mean DR_PBS95%_ in high dose region of 86 Gy/s in the current study versus 185 Gy/s in Sørensen et al. (4)) and a maximum instantaneous dose rate that was only around 300 Gy/s while it was above 1000 Gy/s in Sørensen et al. (4). That we see similar FLASH effects in both studies indicates that the observed FLASH effect may be relatively insensitive to both the PBS dose rate and the instantaneous dose rate as long as the field dose rate (i.e. the mean dose rate) is sufficiently high. For lower field dose rates, the FLASH effect for a given field dose rate depends much more on the detailed beam time structure, as previously demonstrated for this mouse model (30, 46).

Radiosensitivity is sex-dependent and increases with age (47). Thus, care was taken to minimize heterogeneity in the mouse cohort by using single-sex mice within a narrow age interval. This limits the results from a more general conclusion across sex and age, but it was chosen to reduce noise in the data. Another limitation is that the animals were awake during irradiation and thus were able to attempt movement. While the fixation method ensures no major movement, smaller movements could slightly influence the dose delivered to the foot. Our experience is that the movement is minimal and of no major influence, but to mitigate possible influence in the longer delivery of a conventional dose, we used repainting for all conventional groups. Still, the extent of this potential problem was investigated using an *in vivo* scintillator dosimeter attached to the foot, with results published elsewhere.

Future proton radiotherapy could benefit from the implementation of FLASH. The combined SOBP FLASH reduce skin toxicity and fibrotic development, at least compared to a single fraction CONV treatment. As this is not a complete picture of current clinical practice, data from different tissue types and endpoints and fractionated experiments are still needed prior to a broad clinical implementation of FLASH. However, if the same biological response is seen for other tissue types, and the FLASH-sparing effect is conserved with fractionation, SOBP FLASH could enable better radiotherapeutic treatment. A comparison to a fractionated conventional scheme would further facilitate a transition to clinical applicability. Proton radiotherapy can be combined with FLASH using current clinical practice methods of SOBP irradiation and shows excellent potential to reduce side effects, thus improving current treatments.

Our preclinical study quantified the FLASH effect with two robust toxicity assays, yielding a 40% dose-modifying effect for acute skin damage and 18% for late fibrotic damage. The results confirm that SOBP FLASH has a tissue-sparing effect. Our study supports that proton radiotherapy could benefit from using FLASH in a spread-out Bragg peak for the reduction of side effects.

## Data availability statement

The raw data supporting the conclusions of this article will be made available by the authors, without undue reservation.

## Ethics statement

The animal study was approved by Danish Licensing Authority no. 2022-15-0201-01110. The study was conducted in accordance with the local legislation and institutional requirements.

## Author contributions

LK: Writing – original draft, Writing – review & editing, Data curation, Formal analysis, Investigation, Methodology, Validation, Visualization. PP: Writing – review & editing, Conceptualization, Formal analysis, Funding acquisition, Methodology, Project administration, Supervision, Validation, Writing – original draft. EK: Writing – review & editing, Data curation, Formal analysis, Methodology. SR: Writing – review & editing, Data curation, Formal analysis, Methodology, Visualization. CA: Writing – review & editing, Data curation, Formal analysis, Methodology. CEA: Writing – review & editing, Data curation, Formal analysis, Methodology, Writing – original draft. JJ: Writing – review & editing, Conceptualization, Methodology, Project administration, Supervision. YS: Writing – review & editing, Methodology, Validation. UW: Writing – review & editing, Methodology, Validation. CG: Writing – review & editing, Conceptualization, Funding acquisition, Project administration. BS: Writing – original draft, Writing – review & editing, Conceptualization, Funding acquisition, Investigation, Project administration, Resources, Supervision.
