# Supplementary material for: Spread-out Bragg peak FLASH: quantifying normal tissue toxicity in a murine model
Source: Front Oncol. 2024 Jul 3;14:1427667. doi: 10.3389/fonc.2024.1427667 (PMC11256197; doi:10.3389/fonc.2024.1427667)
Supplement: Supplementary file 1 [file Image_1.pdf]

# Supplementary 1

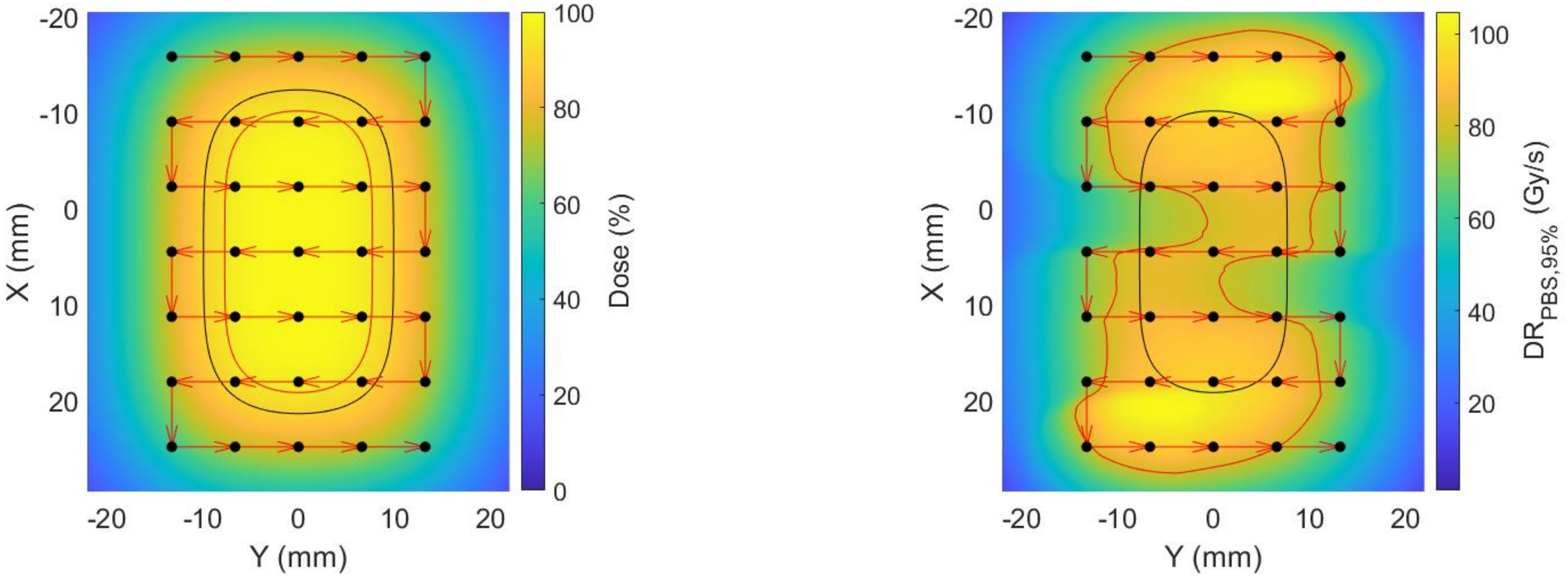


Supplementary 1: 2D dose and PBS (95%) dose rate distribution from spot pattern. A) 2D dose distribution with 95% (red) and 90% (black) isodose curves. B) 2D $DR_{PBS95\%}$ distribution with 95% isodose curve (black) and 80 Gy/s PBS dose rate curve (red)

# Supplementary 2:

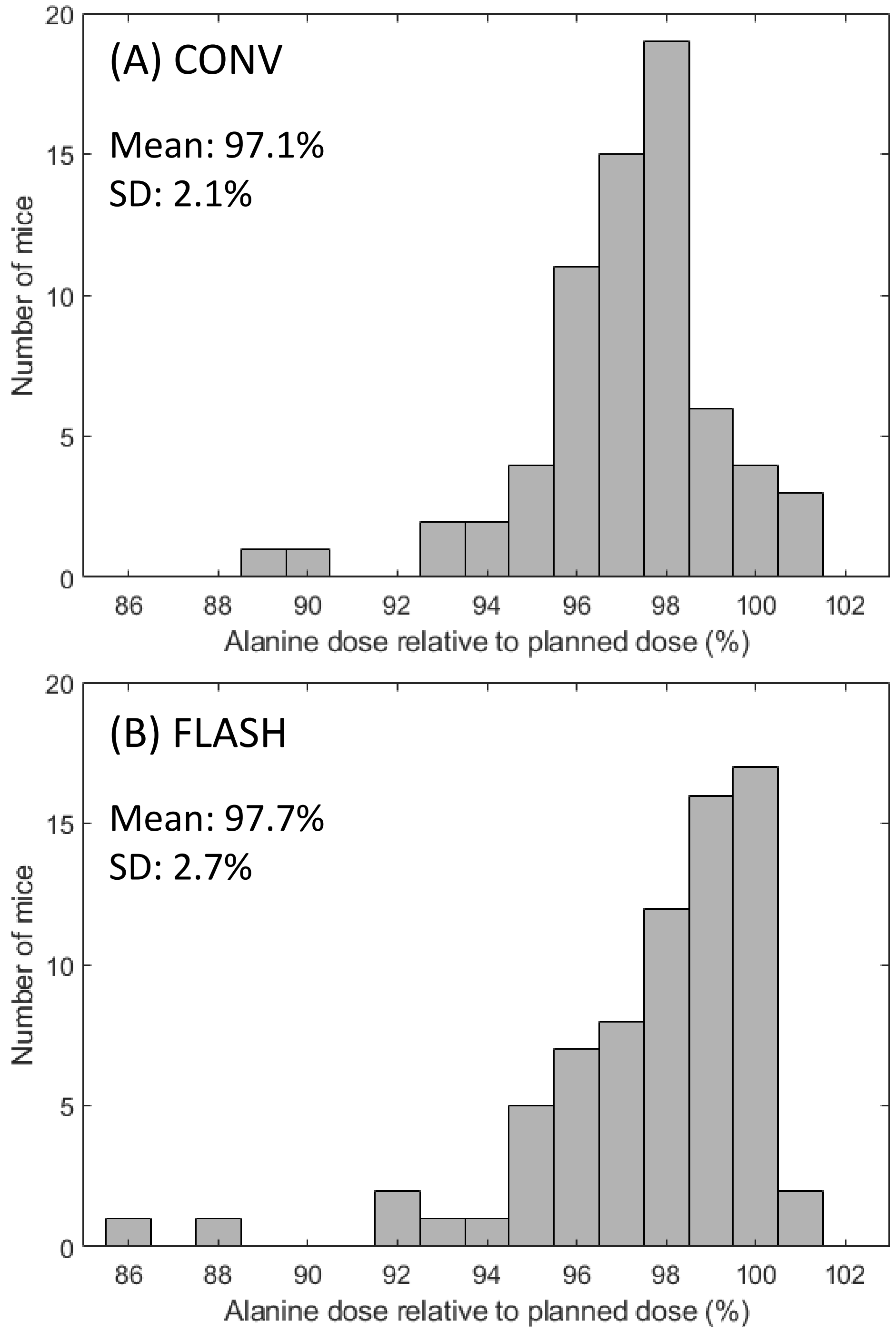


Supplementary 2: Alanine dose distribution of included data for A) conventional (CONV) dose rate and B) FLASH dose rate

# Supplementary 3:

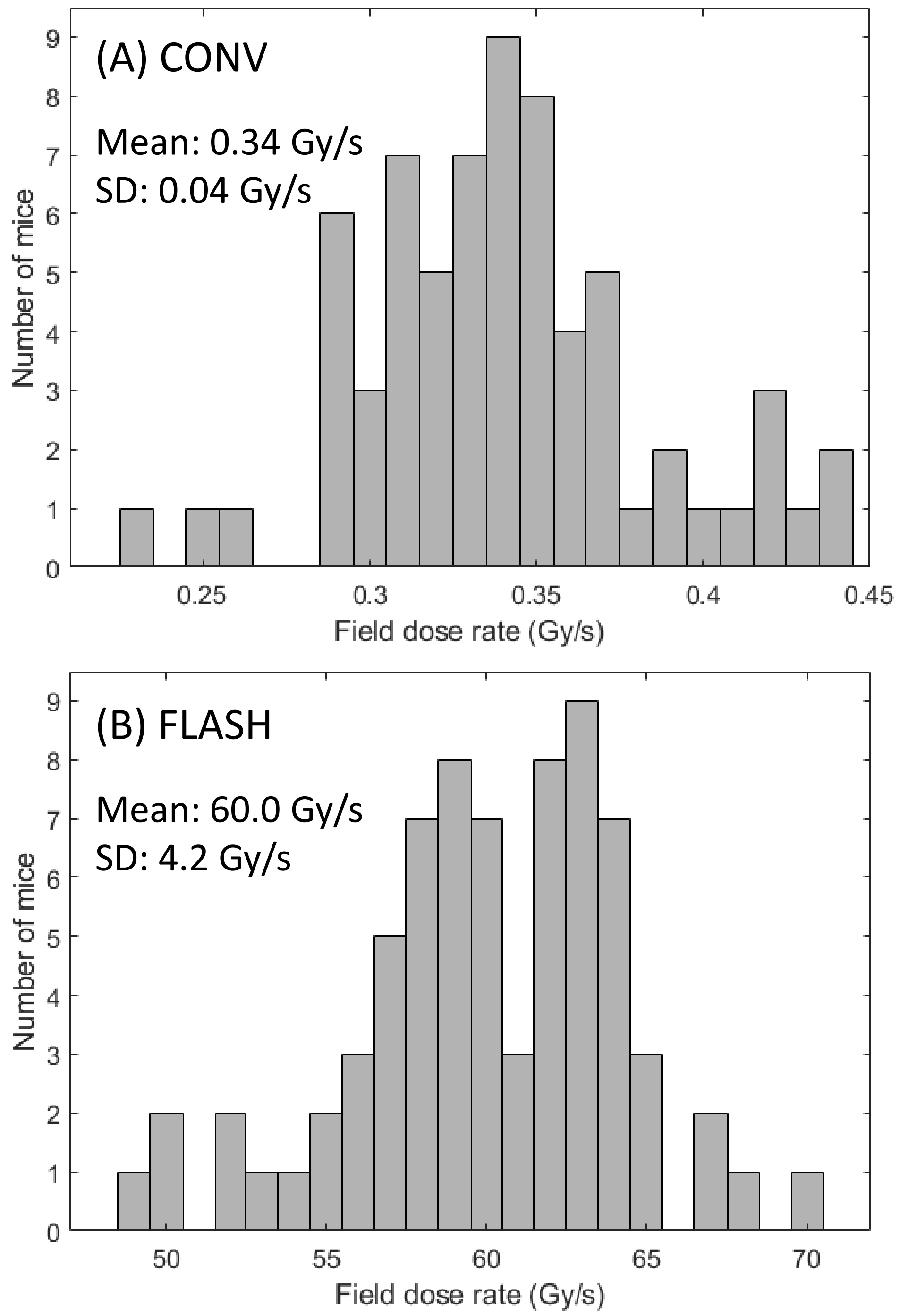


Supplementary 3: Field dose rate distribution for A) the conventional dose rate group and B) the FLASH dose rate group

Supplementary 4:

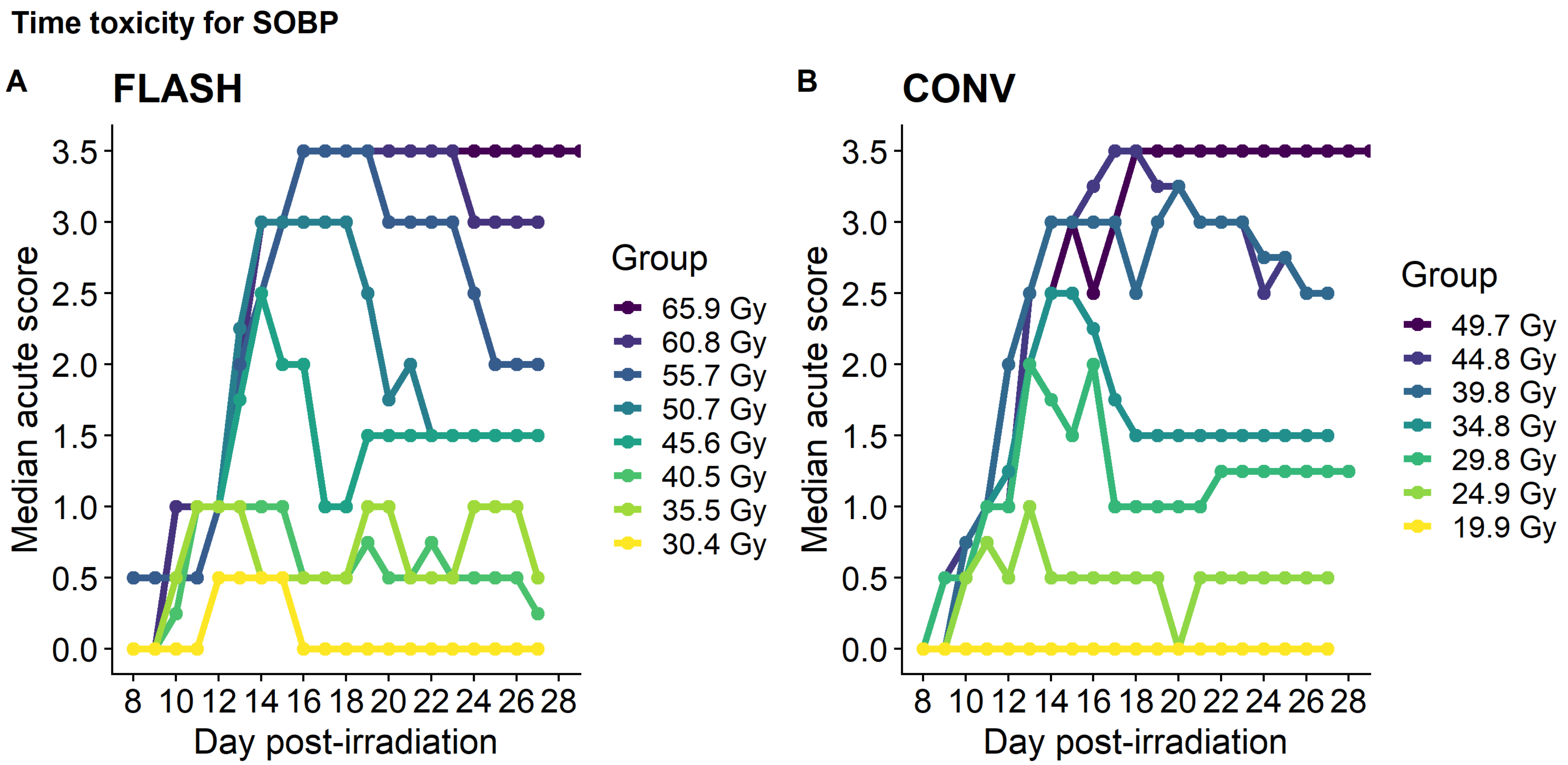


Supplementary 4: Time-dependent development of median acute toxicity for varying doses in A) the FLASH dose rate group and B) the conventional (CONV) dose rate group.
